# The parental co-immunization hypothesis: An observational competing risks analysis

**DOI:** 10.1038/s41598-019-39124-2

**Published:** 2019-02-21

**Authors:** Miguel Portela, Paul Schweinzer

**Affiliations:** 1 NIPE, Universidade do Minho, Campus de Gualtar, 4710-057 Braga, Portugal; 2 Alpen-Adria-Universität Klagenfurt, 9020 Klagenfurt, Austria

## Abstract

The main interest of this study is the hypothesis that contact with small children may be beneficial for the parents’ later health and mortality (because of changes in their immune system). For this purpose, we document the relationship of a set of individual characteristics—including parenthood and marital state—and socioeconomic status with an individual’s cause of death. Using a novel and rich data set made available by the Office for National Statistics Longitudinal Study (ONS-LS), which follows 1% of the population of England and Wales along five census waves 1971, 1981, 1991, 2001, and 2011, our competing risks analysis yields several striking results: (1) Females with children have a 72.5% reduced risk of dying of cancer compared to childless females (for childless females of age 70, this corresponds to a risk of dying of cancer of 1.3% compared to a risk of about 1.3 × 0.275 = 0.4% for females with children). (2) Males have a 171% increased chance of dying of cancer when they are married (e.g., a baseline probability of 1.2% when 75 year old) compared to unmarried males. (3) Females with children have only a 34% risk of dying of heart disease (corresponding to a conditional probability of 0.3% when aged 65) relative to females without children and (4) a 53% chance of dying of infections (i.e., 0.1% at 65 years of age) compared to the risk for females without children. (5) At the same age, married men have an increased expectation of 123% of dying of heart disease (corresponding to an expected death probability of 0.7%) compared to unmarried men. (6) High income and house ownership is always associated with higher survival but less so than having children. While these results document a relationship between the presence of children and mortality, the specific transmission mechanisms remain unclear and we cannot make causality assertions.

## Introduction

The “parental co-immunization hypothesis” is the idea that a parent’s immune system is refreshed by a child’s infections at a time when their own protection starts wearing thin. With this boosted immune system, the parent has a better chance to fend off whatever infections might strike when old and weak and parenthood is rewarded in individual terms through an improved immunization against infections. We conduct an observational competing risks study on individual causes of death and indeed find a reduced parental risk of dying of infections. Similarly, we investigate the relationship of mortality with socioeconomic variables such as house-ownership and income. Since we can document a comparably beneficial association of parenthood with other causes of death which are not compatible with the hypothesis (and therefore cannot be explained by the theory), we also acknowledge the presence of other, perhaps behavioral, factors in parents which result in changed mortality compared to individuals without children.

While the relationships of lifestyle choices such as smoking, obesity, drinking, and other behavioral factors with life expectancy and causes of death are well-studied and understood^1–3^, the same cannot be said for the individual decision to become a parent. The classic theories of aging balance longevity with reproduction and typically predict that, over a genotype’s life span, there is a genetic trade-off between (early) reproduction and late fitness^4^. Hence, these theories usually associate an increased number of children with *decreased* lifespan^5–8^.

The recent literature which is most closely related to the spirit of this paper is Modig *et al*.^9^, who study the association between parenthood and longevity by following Swedish men and women born between 1911 and 1925. They find (in age progressively) lower death risks for individuals having at least one child, than for childless men and women. The study performs sensitivity analyses with respect to the gender of the child, parents’ educational achievements, and geographical distance between parent and child and attributes the difference in death risks to the support children provide to their aging parents. Extending this approach, we are interested in the correlations of an individual’s choice of parenthood and economic status with the causes of death. Zeng *et al*.^10^ is a dose-response meta-analysis of 18 cohort studies including 2.813,418 participants. They provide a detailed comparative overview of the literature and find that moderate-level parity is inversely associated with all-cause mortality: participants with no live birth have a 19% higher relative risk of all-cause mortality compared with participants with one or more live births. Another related and recent observational study is Shadyab *et al*.^11^, which discusses the relationship between children and longevity, finding that women who have their first child after the age 25 are 11% more likely to survive into their 90s than women who give birth first at younger ages. They conjecture that later childbirths reflect better health and do not represent a cause for greater longevity. They summarize the relevant literate on the association of later childbearing with subsequent health outcomes^12^. Similarly related is the investigation of parity in relation to mortality, studying whether the number of offspring has an association with the life expectancy from different diseases. Dior *et al*.^13^ analyze the association between the number of children (i.e., parity) and the mortality of mothers. They observe a U-shaped relationship between the number of offspring and risk of all-cause mortality in mothers, even after accounting for demographic, lifestyle, and reproductive factors. They present no comparative results on women without children. There are several further recent investigations of the association of all-cause or cause-specific mortality with parity that are indirectly relevant to our project^14–16^.

Berntsen^17^ is an investigation into the association of cause-specific mortality with marital state in Norway. In tune with the previous literature, the paper finds that, “relative to married persons, those who are never married, divorced or widowed have significantly higher mortality for most causes of death.” Kaplan and Kronick^18^ study the relation between marital status and survival and Manfredini *et al*.^19^ is a more focused investigation into the association of marital status and disruption with poor physical health outcomes, including all-cause mortality.

A distinct perspective on the relationship between immunization and parenthood is taken by Carr *et al*.^20^, who study the immune systems of adults to understand the main sources of their variations. Their focus is on cohabitation and they find that one of the main factors compressing the variation between individuals’ immune systems is whether or not they share an environment as parents. Further work documenting the human immune system’s responsiveness to environmental exposure includes Kau *et al*.^21^ and Brodin and Davis^22^. Similarly, the “Hygiene Hypothesis” posits that early childhood exposure to germs may help strengthen the immune system and protect children from developing allergies and other illnesses^23,24^.

Marital and economic status variables work as control variables and, to some degree, aim at controlling for selection bias. By including these regressors we attempt to filter out characteristics that in other studies were associated with different death incidence, and, as such, isolate the single effect of having, or working, with children. Our interpretation and discussion of the co-immunization hypothesis is conditional on these socioeconomic status indicators; their association with mortality provides a critical perspective on our hypothesis.

Besides the novelty of employing a competing risks survival analysis for our results, and the use of a rich and sizable data set spanning more than 50 years in 5 UK census waves, the main novelty contributed by this paper is the light shed on the association of human mortality with individual characteristics—such as the decision of having children or getting married—and socioeconomic status variables—such as house ownership or income. As a potential pathway explaining some of our findings, we introduce a new hypothesis on parental co-immunization and test it using the developed competing risks methods. The prime limitation of our study is that it is observational, and it is important to understand that the relationship between the various explanatory variables and mortality could result from selection, not causality.

## Results

To test our hypothesis on the determinants of ‘age of death’ we estimate a set of competing risks models. A discussion of the estimation strategy is provided in the following section. Tables 1 and 2 show results that were estimated using the competing risks model, separately for females and males. A value of 1.0 (i.e., a baseline of 100%) would indicate no effect. Values set **bold**, with the first digit underlined, show a risk-reduction of more than one third. All significant values greater than 1 are set *italic*, with the first digit bold, and correspond with significantly increased risks.

**Table 1:** Contribution to Cause of Death — Female sample. Estimates for ‘Errors and open cases,’ corresponding to 8,380 observations, are not shown.

Variable	Infection	Pulmonary	Cancer	Heart disease	Acc/Hom/Suic	Others
*Failed*	*2*,*254*	*1*,*931*	*8*,*912*	*6*,*770*	*783*	*4*,*904*
yngkids	**0**.528*** (0.028)	0.933 (0.063)	**0**.275*** (0.007)	**0**.344*** (0.010)	**0**.380*** (0.034)	0.735*** (0.030)
working with yngkids	0.746** (0.107)	**0**.643*** (0.111)	0.955 (0.060)	0.877 (0.071)	0.789 (0.178)	0.836** (0.072)
married	0.751*** (0.047)	1.056 (0.082)	***1***.176*** (0.042)	1.066 (0.043)	**0**.565*** (0.054)	0.819*** (0.036)
highclass track	0.689*** (0.032)	**0**.645*** (0.033)	0.721*** (0.017)	**0**.588*** (0.016)	0.694*** (0.055)	0.807*** (0.025)
house owner	0.735*** (0.036)	**0**.606*** (0.031)	0.726*** (0.019)	0.704*** (0.020)	0.797*** (0.067)	0.911*** (0.031)

Reported parameters are subhazard ratios; for each column, the heading indicates the main risk, all others are aggregated into a single competing risk. (Robust standard errors are in parentheses). Significance levels: 10%*, 5%**, 1%***. **Bold** indicates a reduced hazard of more than one third; significant increased risk is shown *italic*. See the following section for a discussion of the variables, the sample and the estimation design. Source: Own computations based on ONS-LS.

**Table 2:** Contribution to Cause of Death — Male sample. Estimates for ‘Errors and open cases,’ corresponding to 10,296 observations, are not shown.

Variable	Infection	Pulmonary	Cancer	Heart disease	Acc/Hom/Suic	Others
*Failed*	*2*,*578*	*2*,*471*	*9*,*704*	*13*,*460*	*1*,*528*	*4*,*804*
yngkids	**0**.654*** (0.034)	1.008 (0.060)	**0**.325*** (0.008)	**0**.385*** (0.001)	**0**.444*** (0.028)	0.778*** (0.031)
working with yngkids	0.677** (0.121)	**0**.488*** (0.110)	0.796*** (0.067)	0.923 (0.060)	0.909 (0.186)	1.103 (0.105)
married	0.753*** (0.043)	0.860** (0.054)	***1***.707*** (0.060)	***1***.230*** (0.035)	**0**.437*** (0.027)	0.669*** (0.028)
highclass track	0.735*** (0.032)	**0**.650*** (0.029)	0.791*** (0.018)	0.807*** (0.015)	0.776*** (0.045)	0.969 (0.030)
house owner	0.806*** (0.038)	**0**.614*** (0.028)	**0**.652*** (0.016)	**0**.661*** (0.014)	0.664*** (0.042)	0.827*** (0.029)

Reported parameters are subhazard ratios; for each column, the heading indicates the main risk, all others are aggregated into a single competing risk. (Robust standard errors are in parentheses). Significance levels: 10%*, 5%**, 1%***. **Bold** indicates a reduced hazard of more than one third; significant increased risk is shown *italic*. See the following section for a discussion of the variables, the sample and the estimation design. Source: Own computations based on ONS-LS.

Hence, the value 0.528 in the first line yngkids of Table 1, column Infection, indicates that the hazard of dying for those with young kids is only 52.8% of the hazard of females without kids. Moreover, all remaining characteristics in the first (Infection) column are also associated with a lowered risk of dying, ranging from 69% to 75%. Thus, the effect of children has roughly twice the magnitude of the other lifestyle variables listed below. All results in the column are statistically significant. The observed beneficial effect of children is highest for cancer (72.5%) but is also strong and significant for all other categories except for pulmonary disease.

A similarly beneficial effect can be seen in the line working with yngkids, our control group consisting of those who work with young children. Being married is generally beneficial when significant. A surprising result is, however, that females are associated with a roughly 18% increase in death risk by cancer if they are married (when compared to non-married females). The status and income variables highclass track and house owner exhibit the expected positive regularities.

Qualitatively similar results are documented in Table 2 for males. Two noteworthy differences are the fact that for Pulmonary disease, being married is associated with a 14% decrease of death risk compared to unmarried males (the corresponding variable is insignificant for females). Also for Cancer, working with children lowers male death risk by 20% (while females are not distinguished along this dimension).

**Figure 1:**
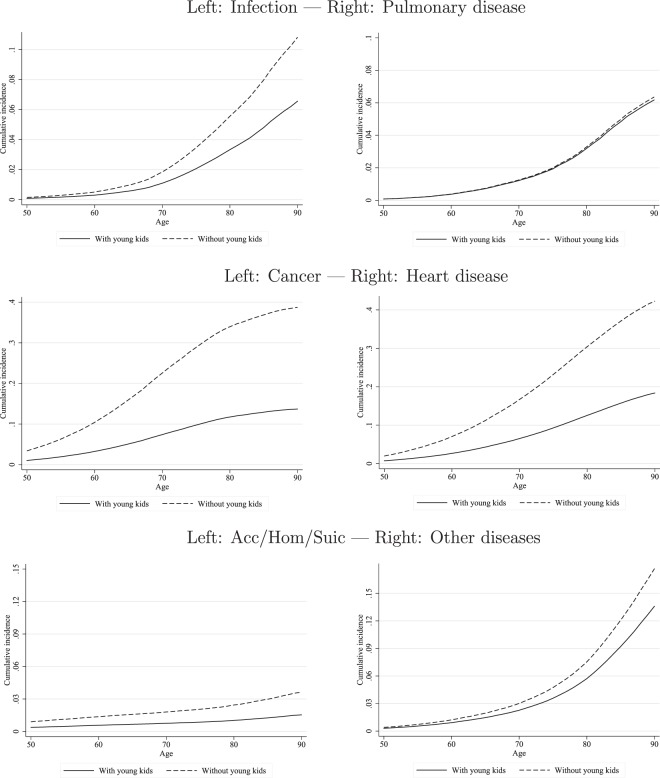
Competing-risks regressions: Cumulative Incidence Functions. Source: Own computations based on ONS-LS.

Figure 1 illustrates the cumulative incidence functions (CIFs) built from the competing-risks regression results for the combined samples of males and females. With the exception of Pulmonary disease, we see the consistent result that the CIF of those with young children show a lower risk of dying at every age. The relative vertical distance between the lines exhibits a natural correspondence with the parameters estimated with the competing-risks models.

## Discussion

The parental co-immunization hypothesis primarily predicts a reduced risk of death from Infection. By themselves, the results shown in the Infection columns of Tables 1 and 2 and the corresponding graphs of Fig. 1 are compatible with the *causal* explanation suggested by the parental co-immunization hypothesis. The demonstrated association of children with most other causes of death, however, cannot be explained by the hypothesis. Since the hypothesis is incompatible with transmission mechanisms which do not rely on immunization, other mechanisms must be present. These may include, for instance, behavioral effects, economic constraints, and other biological influences which an observational study cannot causally disentangle from a co-immunization hypothesis. Hence, the parental co-immunization hypothesis is very likely not the sole causal explanation of the beneficial effect of children on death risks. Nevertheless, our observed results do not reject the possibility of *some* causal influence in the form of a co-immunization hypothesis.

For some causes of death, the correlation with children differ markedly (for both females and males) from the correlation with the socioeconomic variables marital status, income, and house ownership. (The correlation between the socioeconomic variables and mortality is well-documented^25–28^. For the equally well-documented selection and protection effects of marriage, see^29–31^). For instance, sharply opposing effects arise for Cancer and Heart disease between children and married for both sexes. These suggest significant behavioral changes induced by children—perhaps including healthier behavior^32,33^—the effects of which exceed the beneficial health effects of income and status^34^. By comparison, the effect of marriage on mortality is *negative* in these cases of Cancer and Heart disease, which could be attributable to both a more sedentary and dissolute lifestyle adopted by those married without kids^35,36^.

The recent literature on parity derives a U-shaped association of mothers’ number of children with mortality^13^. In line with this observation, we conjectured that the number of children would strengthen the observed effect. However, our empirical exploration has led us to conclude that the relevant factor is whether or not an individual has (had) children, not their number. (When regressing on the number of children, our results became statistically insignificant). This result suggests that all immunization benefits are already arriving with the first child and little additional co-immunization benefit is obtained from exposure to additional infections. This could, for instance, be rationalized with a high correlation among infectious spells experienced by children.

These results on relative risks are broadly in line with simple (unconditional) descriptive statistics which show that, among those who have died of Infectious diseases, females die on average at age 75, while males reach only an average age of 72 years. Similar computations show that the average age of death from an Infectious disease for those who Lived (Worked) with children is 75 (78), compared with 70 (73) for those who never Lived (Worked) with children. This is to say that, among those who died of an infectious disease, females and those who lived or worked with children, die later. A similar pattern is observed for other causes of death, namely, Pulmonary, Cancer or Heart diseases.

### Limitations of this study

(1) Our study is observational, which limits the potential for identifying causal relations between the covariates we analyze and mortality. (2) Among the set of other determinants of death that might be at play, individuals’ self-selection presents an important source of potential bias. For instance, potential (unobserved) determinants of bad health may also be factors that select people to remain unmarried, not to have children, low socioeconomic positions, and early death. (3) We ignore the role unobserved heterogeneity may have in our survival analysis. Although technically unsatisfactory, the lack of alternatives leaves this the only feasible choice. (4) Ethical issues and data availability restrict the set of feasible identification strategies. In particular, the possibility for randomized experiments (potentially able to circumvent the self-selection bias) seems limited. (5) A high fraction of about 9% of the sample’s death causes is designated ‘Errors, Open, Others.’ Although this should not interfere with the competing risks estimates for the other specific causes (as those errors go into the alternative set of risks), this may limit the scope for interpretation of our results.

## Data and Methods

### Data

We use census data from England and Wales provided by the Office for National Statistics Longitudinal Study (ONS-LS) which follows one percent of the respective populations along five census waves 1971, 1981, 1991, 2001, and 2011. From this data set we use information on age (age), time & cause of death (for further information on the full ICD-coded hierarchy which we used to build our seven alternative causes of death in Table 3, see Office of National Statistics^37^), child births (yngkids), marital status (married), profession, income & class status (highclass track), home ownership (house owner), and gender. (For easier identification we use the names in the data set monospaced). From the professional information we identify individuals working with children (working with yngkids) as control group. We define the variable female = 1 for females; it takes the value of 0 otherwise. The precise descriptive statistics are provided in Table 4.

**Table 3:** Health status. Status indicating alive, or cause of death.

Status	Females (%)		Males (%)		Frequency	Share (%)
Alive	65.9	52.3	57.2	47.7	125,502	61.4
Infection	2.3	46.6	2.5	53.4	4,832	2.4
Pulmonary disease	1.9	43.9	2.4	56.1	4,402	2.2
Cancer	9.0	47.9	9.3	52.1	18,616	9.1
Heart disease	6.8	33.5	12.8	66.5	20,230	9.9
Acc/Hom/Suic	0.8	33.9	1.5	66.1	2,311	1.1
Other diseases	4.9	50.5	4.6	49.5	9,708	4.8
Errors, Open, Others	8.4	44.9	9.8	55.1	18,676	9.1
	100.0	48.7	100.0	51.3	204,277	100.0

Within each pair of columns, Females & Males, the left column represents the share of each status, while the right column shows the share of females and males with this specific attribute. Source: Own computations based on ONS-LS.

**Table 4:** Descriptive statistics.

Variable	Females (%)	Males (%)	Overall (%)
age	67.2	65.5	66.3
	(13.1)	(12.9)	(13.0)
yngkids	90.9	88.1	89.5
working with yngkids	4.9	2.5	3.7
married	90.5	88.1	89.3
highclass track	64.1	52.9	58.3
house owner	80.9	79.9	80.4

The total number of observations is 204,277 (the share of females is about 49%). Age is computed in years. (Standard deviations in parentheses). Source: Own computations based on ONS-LS.

The actual data we use has the following characteristics. We start with 788,558 observations of individuals which we restrict to 547,957 by keeping only those alive in 1971. We also drop visitors and perform several consistency checks on the data. From this set we follow the subset of individuals who were alive and aged between 16 and 50 in 1971. With respect to this choice, we advocate that: (1) previous generations had children at younger ages and a lower life expectancy. (2) 16 years is both the definition of full-time minimum working age and (3) the legal minimum age to enter into marriage in England and Wales. (4) We did not want to eliminate individuals for whom one could observe the effect of young children on death incidence (following our immunization hypothesis): an individual who was 16 years old in 1971, if alive, would be 56 in 2011 and was, therefore, exposed to significant mortality risk. The age composition of the sample is not significantly affected by the age restrictions we make: in 1971 about 95% of those present are 18 or older (87% are 21 or older); the average age is about 34 years (with a standard-deviation of 11 years).

For this group of individuals we determine whether or not they have lived (and/or are still living), or have worked (and/or are still working) with children throughout the individuals’ presence in the data and record, if applicable, their reason of death. The combined sample is 204,277 individuals consisting of 99,520 females and 104,757 males (from starting figures of 403,968 and 384,590, respectively).

The ONS-LS data set uses International Classification of Diseases (ICD) codes to categorize the main and, if applicable, contributory reasons of death. These codes come in several revisions of which 8, 9, and 10 are relevant for the census waves we study; for details see World Health Organization^38^. The exact definition of infectious disease we use, for instance, is the following combination of ICD-9 codes (and their earlier and later equivalents): Infectious Diseases 001–139, Chronic Obstructive Pulmonary Disease 490–496, Occupational or Environmental Lung Disease 500–508, Other Diseases of Respiratory System 510–519. The other reasons for death listed in our tables are defined similarly according to the ICD system.

Statistic details on the causes of death are provided in Table 3. In this sample, roughly 39% of the individuals have died. The most common cause of death is Heart disease (25.7%), followed by Cancer (23.6%). Infection attributes to about 6.1% of deaths. Females represent about 49% of the sample being over-represented among those who are Alive. By contrast, they are clearly under-represented in Heart disease (34%) and Acc/Hom/Suic (34%).

From Table 4 we observe that females live on average by about 1.7 years longer than males. Roughly 90% of our sample have young children, yngkids. The share of females who have at some point in their lives worked with young children, working with yngkids, is 4.9% (2.5% for males).

### Design of the empirical strategy

Our event of interest is ‘Death’ associated with a set of specific causes or diseases. Death is most adequately modeled as the probability of dying given that the person survived until that time and, hence, time until failure (duration or survival) models are most appropriate. In the following discussion we assume that time is described by a continuous random variable.

For some subjects in our sample the total survival time cannot be accurately determined. Although for part of the sample the duration data has information on the time from a well-defined starting point until the event of interest occurs, it is also the case that for specific individuals we only know the time until the end of the data collection process. This could happen because the subject drops out (i.e., leaves the study due to some choice), is lost to follow-up (signifying an issue with the ability to follow the individual in the data set), or because the study ends before the subject experiences the event of interest. In the latter case, the individual survived at least until the end of the study and we face right censoring as the individual is removed from the study before the event occurs.

Each individual is characterized by (1) survival time or spell, measured in years, (2) status at the end of the survival time (event occurrence or censored), and (3) the study group (s)he is in. In our case the study groups are ‘Alive,’ dead due to a ‘Specific cause’ (analyzed in a given regression) and dead for ‘Other reason.’ The specific causes of death we consider are Infections, Pulmonary disease, Cancer, Heart disease, Accidents/Homicides/Suicides (Acc/Hom/Suic), and Other causes. We split the analysis into a sequence of steps, where, in each step, we classify each one of those six causes as a ‘Specific cause,’ while aggregating all other causes under ‘Other reason.’

One can interpret the group ‘Other reason’ as a competing risk that occurs instead of the failure event of interest. One needs to specifically deal with different competing events, which implies that the model has to account for the fact that the number of failures from any competing risk (of failure) will condition on the number of failures from the main failure, which, in turn, implies changes in the estimated failure probability. Failures from any competing risk reduce the number of individuals at risk of failure from the cause under analysis^39^. This implies that we cannot treat it as censored, which renders a one-risk-type model, like for instance the Cox model, infeasible to deal with our survival analysis. As such, a competing risks framework becomes a natural candidate for our estimation strategy.

The covariates we consider as possible determinants of ‘Age of Death’ correspond to those usually discussed in the literature. Our analysis uses the following set of explanatory variables: yngkids, working with yngkids, married, highclass track, and house owner (see the previous subsection for a description of the data used in our empirical analysis.) We omit individual education from our analysis (although such information is present in the data) because we conjecture that our model captures the influence of education already through house owner and highclass track, respectively. This seems to be confirmed by the economic literature^40^ that documents significant correlation of education with an individual’s occupation, the variable that we use to build the highclass dummy. We therefore conjecture that the main causality path from education to the inherent behavioral changes that may have an effect on an individual’s health status are mostly subsumed in these already-present control variables.

#### Formulation of the competing risks model

In a general setting, for each individual in a competing risks model, the type of failure is identified by the index *j*, where *j* = 1, …, *k*. The random duration variable is defined by *T*^(*j*)^, where *T*^(*j*)^ is the time to exit/failure to state *j* after the elimination of all other possible states. A spell ends when individuals leave for one of the *k* possible states. The states are mutually exclusive and exhaustive. We assume that there exists only one period of duration.

The *k* random variables, *T*^(1)^, *T*^(2)^, …, *T*^(*k*)^, can be interpreted as latent durations. These are abstract time periods used in the construction of the econometric model underlying our empirical analysis. Entry into a certain state is dictated by the smallest latent time period (the smallest *T*^(*j*)^), so the time to failure can be specified as *T*^(*j*)^ = *min*[*T*^(1)^, …, *T*^(*k*)^]. For each individual, only one *T*^(*j*)^ is observed in the data and others are considered censored. We consider a competing risks model with independent risks under the assumption that the random variables *T*^(1)^, …, *T*^(*k*)^ are independent.

Under this setup it is possible to estimate conditional and unconditional probability functions that characterize the variables *T* and *J*. The expression

1 $${\lambda }_{J}(t,x)=\mathop{\mathrm{lim}}\limits_{dt\to 0}\,\frac{P(t\le T < t+dt,J=j|T\ge t,x)}{dt}$$

is the transition intensity into state j, and *x* is a vector of explanatory variables consisting of individual characteristics. (We design our empirical analysis as single-record data and time-invariant covariates and coefficients. This assertion is a feature of the estimation strategy we have designed. Although individuals are observed over several waves of the census, we have “collapsed” this information into a single observation per individual and recorded their (changing) characteristics over their entire presence in the data. For example, the variable married assumes the value 1 if the individual was ever married in one of the waves he/she is observed in; similarly for the variables yngkids, working with yngkids, highclass track and house owner. By nature, our setup is one of a single spell, as opposed to multiple spells, as the event of interest, death due to various competing reasons, can occur only once).

These functions are designated as cause-specific hazard functions, which can be empirically interpreted as the fraction of survivors at time *t* that subsequently leave for state *j*. (In our survival analysis we ignore the role of possible unobserved heterogeneity due to currently insurmountable technical difficulties in our environment^41^).

Similarly to concentrating on the *cause-specific hazard function*, we also focus on the *cumulative incidence function* (CIF) rather than the survivor function. (The popular Kaplan-Meier statistic would be inadequate for estimating the survival function from lifetime data. Berry *et al*.^42^ summarize the argument: “Kaplan-Meier survival analysis and Cox proportional hazards regression […] can overestimate risk of disease by failing to account for the competing risk of death”). A CIF is just the probability that a specific type of event is observed before a given time, and can be defined as

2 $$CI{F}_{J}(t)=P(T < t,J=j).$$

The CIF gives the proportion of individuals at time *t* who have died of cause *j* accounting for the fact that individuals can die of other causes. For example, the CIF for death due to Infection (which is one of the possible states discussed below) depends not only on the hazard for death by infection but also on the remaining hazards associated with other causes of death. This implies that it is no longer possible to define a direct relation between cause-specific hazard rate and the probability of death.

Although nonparametric estimation of CIFs is flexible, it cannot be adjusted for relevant regressors as they are associated with the cause-specific hazard. The efficient (and correct) way to run CIF covariate analysis is to implement a competing risks regression, according to the model of Fine and Gray^43^. They propose an alternative to cause-specific hazards: a semiparametric model for the hazard of the subdistribution for the failure event of interest, known as the subhazard. The *subhazard function* for failure cause *j* can be defined as

3 $${\bar{\lambda }}_{J}(t,x)=\mathop{{lim}}\limits_{dt\to 0}\,\tfrac{P\{(t\le T < t+dt,J=j|T > t\,{\rm{or}}\,(T\le t\,{\rm{and}}\,J\ne j),x)\}}{dt}.$$

Under this formulation, there is a one-to-one correspondence between subhazards and CIFs for respective event types; that is, the CIF for a specific cause of death is a function of only the subhazard for that cause of death. Covariates affect the subhazard proportionally, similar to the Cox regression. From the relation between the hazard and survival functions, Fine and Gray^43^ define a subdistribution function. (For a more detailed discussion of competing risks models see, for instance, Kalbfleisch and Prentice^44^).

## Data Availability

The data that support the findings of this study were made available to the authors under the Approved Researcher Scheme from the United Kingdom Office for National Statistics (ONS) Longitudinal Study’s Centre. The permission of the Office for National Statistics to use the Longitudinal Study is gratefully acknowledged (clearance #30130), as is the help provided by staff of the Centre for Longitudinal Study Information & User Support (CeLSIUS). CeLSIUS is supported by the ESRC Census of Population Programme (Award Ref: ES/K000365/1). The authors alone are responsible for the interpretation of the data.This work contains statistical data from ONS which is Crown Copyright. The use of the ONS statistical data in this work does not imply the endorsement of the ONS in relation to the interpretation or analysis of the statistical data. This work uses research datasets which may not exactly reproduce National Statistics aggregates.
